# Perioperative smoking cessation in vascular surgery: challenges with a randomized controlled trial

**DOI:** 10.1186/s13063-015-0965-x

**Published:** 2015-10-05

**Authors:** Mette Kehlet, Sabine Heeseman, Hanne Tønnesen, Torben V. Schroeder

**Affiliations:** Vascular Clinic, Rigshospitalet, Blegdamsvej 9, 2100 Copenhagen, Denmark; Vascular Clinic, Lillebaelt Hospital, Søndre Boulevard 29, 5000 Odense C, Denmark; WHO-CC Clinical Health Promotion Centre, Bispebjerg and Frederiksberg Hospital, Nordre Fasanvej 57, 2000 Frederiksberg, Denmark; Clinical Health Promotion Centre, Department of Health Sciences, Lund University, Skåne University Hospital, Södra Förstadsgatan 101, 214 28 Malmö, Sweden; Centre for Clinical Education, University of Copenhagen and Capital Region of Denmark, Blegdamsvej 9, 2100 Copenhagen, Denmark; Faculty of Health and Medical Sciences, University of Copenhagen, Blegdamsvej 3B, 2200 Copenhagen N, Denmark

**Keywords:** Randomized clinical trial, Gold Standard Program, Smoking cessation, Postoperative complications, Peripheral vascular surgery

## Abstract

**Background:**

The effect of intensive smoking cessation programs on postoperative complications has never before been assessed in soft tissue surgery when smoking cessation is initiated on the day of surgery.

**Methods:**

A single-blinded randomized clinical trial conducted at two vascular surgery departments in Denmark. The intervention group was offered the Gold Standard Program (GSP) for smoking cessation intervention. The control group was offered the departments’ standard care. Inclusion criteria were patients with planned open peripheral vascular surgery and who were daily smokers. According to the power calculation a total of 144 patients were needed in the trial.

**Results:**

Due to slow patient inclusion, the trial was terminated prior to fulfilling the power calculation. Thirty-two patients were included in the trial from March 2011 to September 2012. Of these, 11 were randomized to the GSP intervention and 21 as controls. There was no difference in 30-day complication rates or 6-week abstinence rates between the two groups.

**Conclusions:**

A trial assessing the effect of smoking cessation on postoperative complications on the day of soft tissue surgery is still needed. If another trial is to be planned it must be more pragmatic with less extended inclusion criteria and conducted nationally or internationally to ensure enough patients for the trial.

**Trial registration:**

ClinicalTrials.gov (NCT01469091). Registration date: 27 October 2011.

**Electronic supplementary material:**

The online version of this article (doi:10.1186/s13063-015-0965-x) contains supplementary material, which is available to authorized users.

## Background

Smoking is a well-known risk factor for developing complications after surgery. The reason that smoking tobacco is a risk factor for developing postoperative complications is multifactorial. The tobacco and the additives affect most cells and tissues in the body. Wound healing is slower due to diminished oxygenation of the tissues, impaired microcirculation and release of vasoactive components [1, 2]. Electrocardiograms studied during surgery in smokers have shown signs of heart muscle ischemia [3, 4]. Smoking is a well-known risk factor for developing chronic obstructive pulmonary disease (COPD), but even in smokers with normal lung function the tobacco smoke also impairs the movement of the cilia in the bronchioles and bronchus causing stagnation of secretion and with fewer macrophages than in non-smokers, all adding to an increased risk of pneumonia [5, 6].

Three studies have shown that intensive smoking cessation programs 4–8 weeks prior to surgery reduced postoperative complications significantly [7–9]. Even in the acute setting, a significant effect of smoking cessation intervention has been shown on results of acute fracture surgery [10].

The vascular patient population is often perceived as more heavy smokers than the general smoker population and therefore possibly also has increased risk of postoperative complications when in need of surgery. Smoking cessation intervention among vascular surgical candidates might therefore be additionally challenging, but has not previously been reported.

We decided to set up a randomised controlled trial to assess the effect of an intensive smoking cessation program on results of acute vascular surgery. We intended to include patients on the day of admission and to institute the smoking cessation program during the immediate postoperative period. The primary outcome was the occurrence of perioperative complications, and among the secondary outcomes we were particularly interested in whether patients remained abstinent from the day of surgery until 6 weeks after surgery.

## Methods

### Design

The trial was designed as a clinical controlled single-blinded randomized trial. It was registered in ClinicalTrials.gov (NCT01469091), registration date October 27, 2011, and approved by the Danish National committee on Health Research Ethics (H-1-2010-112).

### Setting

This trial was conducted at the two departments of vascular surgery at Rigshospitalet and Gentofte Hospital, Copenhagen, and at Lillebaelt Hospital (former Kolding Hospital). The departments serve as primary referral for vascular diseases of 2½ million people corresponding to 2/5 of the Danish population.

A pilot study from the Danish National Vascular Registry showed that the number of patients fulfilling the inclusion criteria was 144 in the year prior to the project start.

The trial was initiated in March 2011 in Copenhagen for patients admitted acutely and operated on for peripheral arterial surgery within 72 hours of admittance (phase 1). Due to slow patient inclusion the vascular department at Lillebaelt Hospital was enrolled in the project as of August 2011. By January 1, 2012 the project was extended to include also patients admitted for elective peripheral arterial surgery (phase 2).

### Participants

Inclusion criteria were daily smokers admitted for primary open infrainguinal arterial surgery. Initially only patients admitted acutely and operated upon within 72 hours were included, but as described above, patients admitted for elective surgery were included also during phase 2 of the trial.

Exclusion criteria were alcohol consumptions of more than 35 units a week, being pregnant or patients younger than 20 years. Also patients who had been operated upon in the same vascular segment within 90 days were excluded, as well as patients who could not give informed consent.

Patients who had quit smoking within 3 days of admittance were considered smokers and were eligible for inclusion.

### Randomisation

The Section of Biostatistics, University of Copenhagen made a list with randomization numbers, stratified for each of the two departments. The patients were randomized in a 1:1 ratio for the intervention or the control group in blocks of ten. The envelopes used were opaque and sealed and were numbered consecutively. A doctor at the department, who was not involved in the trial, kept the randomization list in a locked drawer until the trial was terminated, all follow-ups had been conducted and data were registered.

The vascular departments’ doctor on duty gave all patients eligible for inclusion oral and written information of the project at the time of admittance. All patients gave a written form of consent before inclusion. The departments’ doctor on duty also performed the randomisation. Whether the participants were randomized to intervention or control group was open to the nursing staff and the doctor performing the randomization, but was blinded for the examining and data-collecting doctor in the outpatient clinic.

At the primary investigators’ department all admittance records were screened several times a week to identify potential participants who had not been informed about the trial.

Within 72 hours after surgery the patients included in the trial were offered supplementary information by the primary investigator or project nurse, to make sure they had received and understood the information on the project.

### Intervention

Patients who were randomized to smoking cessation intervention were offered the Gold Standard Program (GSP) that has been the smoking cessation program of choice in Denmark since 2001. It is an intensive smoking cessation program developed by the National Cancer Society and the National Stop Smoking Centre [11] to improve and standardize smoking cessation programs in Denmark. It consists of a standard program with five meetings over 6 weeks where each meeting is predefined with motivational conversation, smoking habits and education on the hazardous effects of smoking as well as the benefit of successful quitting. Other meetings revolve around risks of relapse, thoughts and reflections on quitting, challenges during and after the course. The first meeting was within 48 hours after surgery depending on the patient and the project nurse. At the first meeting the patients were scored using the Fagerström Test for nicotine dependence [12] and the number of cigarettes smoked per day was recorded. Accordingly, they were recommended the dosage of nicotine replacement therapy, if interested. Together with the smoking cessation instructor they decided which administration form of nicotine replacement therapy could be useful. Nicotine gum, patches, inhalators and microtabs were available for the participants. Nicotine replacement therapy is the primary recommendation for smoking cessation intervention in Denmark [11]. No other medical replacement was offered (i.e., bupropion/varenicline). Normally, the GSP is free of charge but patients must themselves pay for the nicotine replacement and the course is offered as group- or individual-based. We offered free nicotine replacement and individual meetings to all participants randomized for the GSP corresponding to the previous studies on GSP for surgical patients [8–10]. Three nurses from the outpatient clinics, all trained smoking cessation instructors according to the GSP, were responsible for the smoking cessation meetings and patients were classified ‘compliant with program’ if they participated in at least 75 % of the meetings.

Additional to the information about the trial, given by the admitting doctor, the control group was offered the hospital departments’ standard care. This could be anything from no information on smoking cessation to the admitting doctor’s bedside information about the risk of smoking and the benefits of quitting. The control group was also offered free nicotine replacement during admittance as part of the departments’ standard care, but after discharge it was the patients’ responsibility to seek further help to smoking cessation if they wished.

After surgery and discharge all participants were seen in the outpatient clinic for a 6-week follow-up at the end of the GSP, a 3-month follow-up and 1-year follow-up to examine the effect of the operation performed, to register any complications and to assess the participants smoking status.

### Data

After written informed consent was given, data was collected for the case-report file: gender, age, cigarettes smoked per day, type of surgery (acute versus elective), educational level and working status. Patients also answered the short form 36 (SF-36), which is a questionnaire with 36 questions concerning quality of life and health care. Measure points are both physical and mental health [13].

After discharge and at follow-up in the outpatient clinic any complication requiring treatment was recorded. Again the questionnaire SF-36 was filled in and smoking status was obtained. Smoking status was self-reported as well as validated by carbon monoxide breath test.

### Outcome

The primary outcome was occurrence of postoperative complications requiring treatment within 30 days postoperatively. Complications were classified into four groups according to the Danish National Vascular Registry [14]:

- Wound complications (infection, lymphorrhea, necrosis)
- Surgical complications (bleeding, emboli/thrombosis of the reconstruction, peripheral nerve lesion, ileus or ischemia of bowels)
- General complications (cardiac or pulmonary complications, dialysis, transitory cerebral ischemia or stroke, deep venous thrombosis, compartment syndrome, urinary tract syndrome and multiorgan dysfunction syndrome)
- Death ≤ 30 days

Secondary outcomes were:

- Patency of reconstruction, 30 days and 1 year postoperatively
- Amputation rate, 30 days and 1 year postoperatively
- Length of stay (LOS)
- Continuous tobacco abstinence 6 weeks, 3 months and 1 year
- Physical and mental health according to SF-36, 6 weeks, 3 months and 1 year
- Time to return to normal activity

### Statistical analysis

Power calculation of the sample size was performed using the formula: N > (Z_2α_ + Z_2β_)2 × S^2^ /minimum relevant difference (MIREDIF). With 2α = 5 % and 1-β = 80 % and the MIREDIF = 0.2. The power analysis of the sample size assessed that 144 patients should be included in the trial, 72 in the intervention group and 72 in the control group. Analyses of the postoperative results were done by using SPSS version 22 (IBM Corp., Armonk, NY, USA). The variables were assessed using Fisher’s exact test (two-sided).

## Results

Due to lack of patient inclusion, the trial was terminated prematurely prior to reaching the number of patients prespecified in the power calculation. Recruitment for the trial was terminated by the end of September 2012. At that point, we had succeeded in recruiting 21 patients in Copenhagen over 19 months and 11 patients in Kolding over 14 months.

**Fig. 1:**
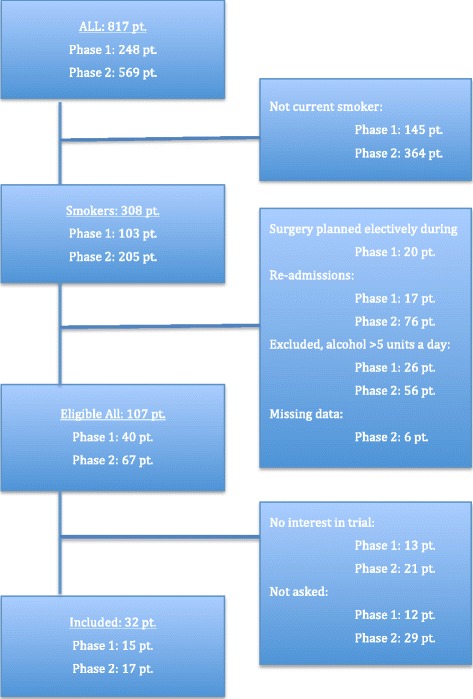
Flow chart for patient eligibility and inclusion. Phase 1: 1 March 2011 to 31 December 2011, acute/subacute admittance. Phase 2: 1 January 2012 to 30 September 2012, acute/subacute and elective admittance

Within the chosen time period, a total of 817 potentially eligible patients were admitted to the participating vascular departments for peripheral arterial surgery. Of these, 248 patients were admitted during phase 1 (acute/subacute) and 569 patients were admitted during phase 2 (acute/subacute and elective) (Fig. 1).

A total of 308 (38 %) were current smokers. However, the following had to be excluded according to the criteria: surgery performed as an elective procedure (20 patients, during phase 1), readmitted and operated upon in the same vascular segment within the previous 90 days (17 + 76 patients), drinking more than 5 units/day (26 + 56 patients), and finally six patients in whom data were missing (Fig. 1).

This left 107 patients eligible for inclusion. A total of 34 patients refused to take part in the trial and another 41 patients were not asked, for logistical reasons. At the department where the primary investigator was working full-time with the trial and screened admittance records several times weekly, 12 patients were not asked throughout the trial period (19 months) due to courses and vacations. At the second department, the investigator was only involved in the project part-time and missed 29 patients throughout the trial period (14 months) due to courses, vacations and because of the work schedule.

**Fig. 2:**
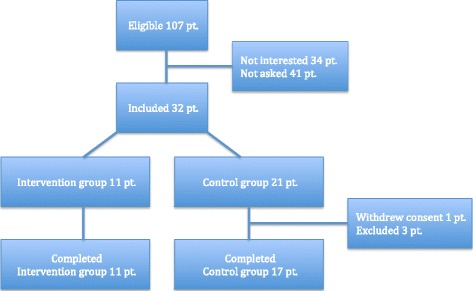
Flow chart for patient inclusion, randomization and completion of the trial

Of the 32 patients recruited for the trial, 11 were randomized to the GSP intervention and 21 were randomized as controls. In the control group, one patient withdrew consent to participate the day after inclusion and three patients were excluded because they had aortic surgery and not infrainguinal surgery. This left 11 patients for GSP intervention and 17 patients in the control group (Fig. 2). For the Consort 2010 checklist and flow diagram, see Additional files 1 and 2.

The median age of the patients in the GSP intervention group was 61 years versus 65 years in the control group. The median number of days in hospital was 9 days for the GSP patients and 7 in the control group. Cigarette status at admittance showed that 64 % smoked more than 20 cigarettes per day in the GSP intervention group compared to only 18 % in the control group (*p* = 0.02). In the intervention group, the preferred nicotine replacement of choice was combined long-term-acting and fast-acting therapy – thus following the general recommendation on nicotine replacement therapy. Overall, nine participants used patches (25 mg/16 hours: six participants, 20 mg/16 hours: one participant, 15 mg/16 hours: two participants). All but one combined with gum, inhalators or microtabs. Only two participants in the intervention group did not choose any nicotine replacement therapy. For the rest of the demographic data see Table 1.

**Table 1:** Demographics for the GSP intervention group and control group

	GSP intervention	Control group
Number of patients:	N = 11	N = 17
Age (years)	61 (39–72)	65 (52–80)
Male gender	9 (82)	13 (76)
Acute operation	6 (55)	8 (50)
Cigarettes per day	22 (10–50)	14 (2–50)
Educational level:		
Missing information	3	3
Primary school	5 (45)	9 (53)
High school/college/university	3 (27)	5 (29)
Occupation:		
Missing information		1
Working	2 (18)	4 (24)
Retired	9 (82)	12 (71)

Data are from time of inclusion, given as number (%) or median (range)

*GSP* Gold Standard Program

The peripheral vascular procedures performed were bypass from the femoral artery to the popliteal artery and branches (intervention/complication group: 46 %/47 %), thrombectomies of the femoral arteries and popliteal artery (9 %/24 %), transendarterectomies of the femoral arteries (27 %/24 %) and femorofemoral crossover bypass (18 %/5 %), see Table 2.

**Table 2:** Surgical procedures performed on the GSP intervention group and the control group

	GSP intervention group N = 11	Control group N = 17
By-pass from the femoral artery and branches to the popliteal artery and branches	5 (46)	8 (47)
Thrombectomy of the femoral artery, popliteal artery and branches	1 (9)	4 (24)
Transendarterectomy of the femoral artery and branches	3 (27)	4 (24)
Femorofemoral crossover bypass	2 (18)	1 (5)

Numbers = N (%)

*GSP* Gold Standard Program

In both groups there were recorded eight complications in seven patients (*p* = 0.44) within the first 30 days. Patients in the GSP intervention group were compliant with the smoking cessation program in 73 % of cases. Smoking abstinence after 6 weeks was achieved in three of 11 (27 %) patients in the GSP intervention group and in three of 17 (18 %) patients in the control group (Table 3). One patient died due to an oral cancer 11 months postoperatively and two patients were transfemoral amputated at 4 and 7 months respectively. All three patients were randomized to the GSP intervention group.

**Table 3:** Six-week follow-up results

	GSP intervention group N = 11	Control group N = 17
Compliant with GSP program	8 (73)	NR
Abstinent from tobacco	3 (27)	3 (18)
Wound complication, 30 days*	5 (45)	7 (41)
Surgical complication, 30 days*	3 (27)	0
General complication, 30 days*	0	1 (6)
Length of stay, days (range)	9 (4–17)	7 (2–30)
Amputation rate	0	0

Numbers = N (%)

*GSP* Gold Standard Program, *NR* not relevant

*For definitions of complications see text

Due to the few patients included in the trial, the secondary outcomes: patency of reconstruction, SF-36 and time to return to normal activities are not reported, and we refrained from statistical testing for any group differences.

## Discussion

It was very disappointing that we eventually had to terminate this trial prematurely. We succeeded in enrolling only 32 of the planned 144 (22 %) patients during a time period of 19 months.

Our observation was that out of 817 patients admitted for acute or elective peripheral arterial surgery only 107 were eligible for inclusion (Fig. 1) and of the 66 patients asked 52 % did not wish to participate, which correlates well with the declining rate of comparable trials (40–62 %) [8, 10]. All of the eligible patients who declined gave more or less the same reason for declining: they wanted to quit smoking but they were not interested in following a smoking cessation program. There were 41 eligible participants who were not asked to participate for logistical reasons such as courses, vacations and work schedules of the investigators.

We ask ourselves whether we could have foreseen the difficulties in recruiting patients for this trial.

In designing the trial, reflections were made on the group of patients included. Patients having open abdominal vascular surgery, i.e., aortic surgery, were excluded due to the different surgical procedures, different profile of postoperative complications and a higher mortality [15, 16] than that of peripheral vascular surgery. Likewise, the increasing group of patients undergoing endovascular procedures was not included, since the surgical trauma is usually of very moderate size and the procedure is done under local anesthesia. Both reduce the risk of postoperative complications compared to open surgery and the complication rate after endovascular procedures is reported about 5–10 % [17, 18]. The number of patients to be included in a corresponding study on endovascular-treated patients would be nearly 1000. Therefore, broadening the inclusion criteria to other surgical procedures would have made the trial much more demanding, we thought.

A reduction of the exclusion criteria was also considered. A relatively large fraction of the patients who smoked consumed more than 5 units of alcohol per day, which per se increases the risk of complications significantly [19–21], and the hypothesis did not include an evaluation of the effect of smoking cessation intervention on alcohol-induced complications after surgery. Thus, including the hazardous drinking smokers was not an option, even though they accounted for almost 27 % of the 308 smokers in the trial. Furthermore, the three previous randomised studies on GSP targeting perioperative smoking also excluded the hazardous drinkers from their study population [8–10].

We also excluded patients who had been operated upon in the same arterial segment within 90 days, but it might have been relevant to reconsider that criterion in spite of their greater risk of postoperative complications [22]. It would probably have required an extra stratification, in order to secure similar distribution in the intervention and control group.

Even if smoking cessation on the day of surgery has been shown to be able to reduce the postoperative complications in orthopedic patients, it cannot be transferred uncritically to the vascular patient population, we still have reason to believe that the vascular patients would benefit from smoking cessation.

Other randomized controlled trials using intensive pre- and perioperative smoking interventions on the effect of smoking cessation on postoperative complications have all shown a significant reduction in complications in the intervention group [8–10, 23]. In our trial the prespecified number of patients was not reached, and therefore it was not possible to show any effect of the GSP intervention. Thus we were unable to assess any effect on the occurrence of perioperative complications between the two groups. Neither could we evaluate on the secondary outcome, whether there was a difference in smoking behavior after 6 weeks. The small number of patients included is the reason why we have chosen not to exercise any further statistical analysis on the data depicted in Tables 1, 2 and 3 and why the results of the SF-36 obtained are not reported.

We found that 27 % of the GSP intervention group as opposed to 18 % of the control group were abstinent during the first 6 weeks after surgery. This is lower than the other surgical studies using the same program showing quit rates of 43–60 % in the GSP intervention group compared to 10–20 % in the control group [9, 10]. Apart from the fact that the power calculation was not met, another reason could be a weaker performance of the GSP in the involved departments. However, all the nurses were trained at the same national program. Cohort studies evaluating the GSP for smoking cessation in real-life settings in Denmark have shown that for heavy smokers the abstinence rate was 33 % 6 months after intervention [24], for unemployed smokers it was 27 % versus 34 % for employed smokers 6 months after intervention [25], and for elderly smokers > 60 years it was 37 % 6 months after intervention [26]. The British nationwide ‘stop smoking services’ found an overall abstinence rate of 36 % 4 weeks after intervention [27]. These abstinence rates correlate well the abstinence rates in the vascular patient population, but the difference between our intervention group and our control group was not noticeable.

There may exist a clinical culture of low expectancy to the vascular patients’ ability to quit smoking successfully, which can influence the outcome significantly. Such a culture is supported by the existence of smoking rooms for patients at some of the hospitals involved. In addition, the widespread smoking in the vascular patient population may play a role; there are 44 % daily smokers [15], corroborating the 38 % found in this trial, and in contrast to the 17 % found in the Danish population, in general [28]. From clinical experience, there is also reason to believe that the vascular patient population is still smoking, in spite of their smoke-related illness, may smoke more heavily than the background population, and are more stigmatized by their smoking. Last but not least, the admitting doctor informed all eligible patients verbally and in writing about the health benefits of smoking cessation according to the protocol. So even if the patients allocated to the control group did not receive the GSP, they still received more information on the benefits of smoking cessation than when normally admitted prior to vascular surgery, and therefore maybe the difference of intervention between the GSP and control groups was smaller than we anticipated.

If we were to set up another trial assessing the effect of smoking cessation on postoperative complications, we would have to reconsider the design of the trial according to the experiences and challenges we faced during the execution of this trial.

As discussed above, our inclusion and exclusion criteria were quite extended, giving the (expected) results high statistical credibility and internal validity.

Designing a future trial more pragmatically could be appropriate. A pragmatic trial would include all vascular patients who smoke admitted for open surgery or endovascular procedures (both aortic, peripheral, carotid, endovascular, re-operations, excessive alcohol consumers, etc.) ensuring a higher number of eligible patients. This would increase the external validity making the results directly applicable to the vascular community. However, such a trial would demand inclusion of a huge number of patients followed for a longer period of time, both adding to the cost of the trial. The larger sample size would necessitate several more centers to be included. Securing the uniformity and quality of the smoking cessation instructors and having devoted full-time primary investigators at each site pose additional challenges, though not insurmountable.

Overall, smokers undergoing open vascular surgery are still at high risk. New research must focus on risk reduction. Based on the trial, we would recommend making sure that the smoking cessation program used is followed by a higher level of successful quitting. Furthermore, it should be a national or international trial ensuring inclusion of the number of patients needed, and even so possibly expand the inclusion criteria and reduce the exclusion criteria to include all vascular patients who smoke.

The trial further inspires to develop an evaluation of a simultaneous alcohol and smoking cessation intervention program for risk reduction at surgery.

## Conclusions

Including patients in the single-blinded randomized clinical trial, aimed to assess whether smoking cessation on the day of peripheral vascular surgery could reduce perioperative complications compared to a control group, turned out to be more challenging than anticipated. Even though inclusion criteria was extended from only acute admitted patients to elective patients as well, it was not possible to reach the patient number needed according to the power calculation.

In the material from the small number of included patients, no difference was seen in the rate of 30-day postoperative complications nor could we show a difference in abstinence from smoking 6 weeks postoperatively between the two groups.

A randomized trial assessing the effect of smoking cessations on the day of surgery in vascular patients is still needed. If a trial were to be completed we would recommend it to be designed more pragmatically and conducted nationally or internationally to ensure eligible patients.

## Additional files

Additional file 1:
**CONSORT 2010 checklist.** Completed checklist for the CONSORT statement (DOC 217 kb) Additional file 2:
**CONSORT 2010 flow diagram.** The CONSORT flow diagram showing the different phases of enrollment, allocation, follow-up and analysis for the trial (DOC 49 kb)

## Abbreviations

COPD: chronic obstructive pulmonary disease
GSP: Gold Standard Program: an intensive 6-week smoking cessation program
LOS: length of stay: days of admittance in hospital
MIREDIF: minimum relevant difference
SF-36: short form 36: a physical and mental health score
