# Supplementary material for: Perioperative smoking cessation in vascular surgery: challenges with a randomized controlled trial
Source: Trials. 2015 Oct 5;16:441. doi: 10.1186/s13063-015-0965-x (PMC4595121; doi:10.1186/s13063-015-0965-x)
Supplement: Additional file 2: — CONSORT 2010 flow diagram. The CONSORT flow diagram showing the different phases of enrollment, allocation, follow-up and analysis for the trial (DOC 49 kb) [file 13063_2015_965_MOESM2_ESM.doc]

**
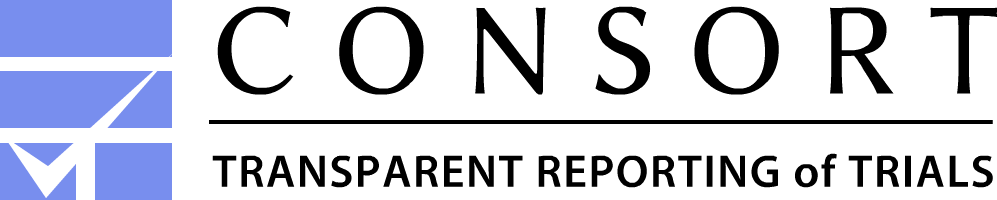
**

**CONSORT 2010 Flow Diagram**

**Allocation**

**Analysis**

**Follow-Up**

**Enrollment**

Assessed for eligibility (n=308 )

Excluded (n=276 )

♦  Not meeting inclusion criteria n=195 )

♦  Declined to participate (n=34 )

♦  Other reasons (n=47 )

Analysed (n=Not relevant )
♦ Excluded from analysis (give reasons) (n=Not relevant )

Lost to follow-up (give reasons) (n= 0 )

Discontinued intervention (give reasons) (n=0 )

Allocated to intervention (n= 11 )

♦ Received allocated intervention (n= 11 )

♦ Did not receive allocated intervention (give reasons) (n= 0 )

Lost to follow-up (give reasons) (n=0 )

Discontinued intervention (give reasons) (n=0 )

Allocated to control (n= 21 )

♦ Received allocated control(n= 17 )

♦ Did not receive allocated intervention (give reasons) (n=4 ) (1 withdrew consent and 3 were excluded)

Analysed (n=Not relevant )
♦ Excluded from analysis (give reasons) (n=Not relevant )

Randomized (n=32 )
